# Supplementary figures and images for: A flexible and generalizable model of online latent-state learning
Source: PLoS Comput Biol. 2019 Sep 16;15(9):e1007331. doi: 10.1371/journal.pcbi.1007331 (PMC6762208; doi:10.1371/journal.pcbi.1007331)

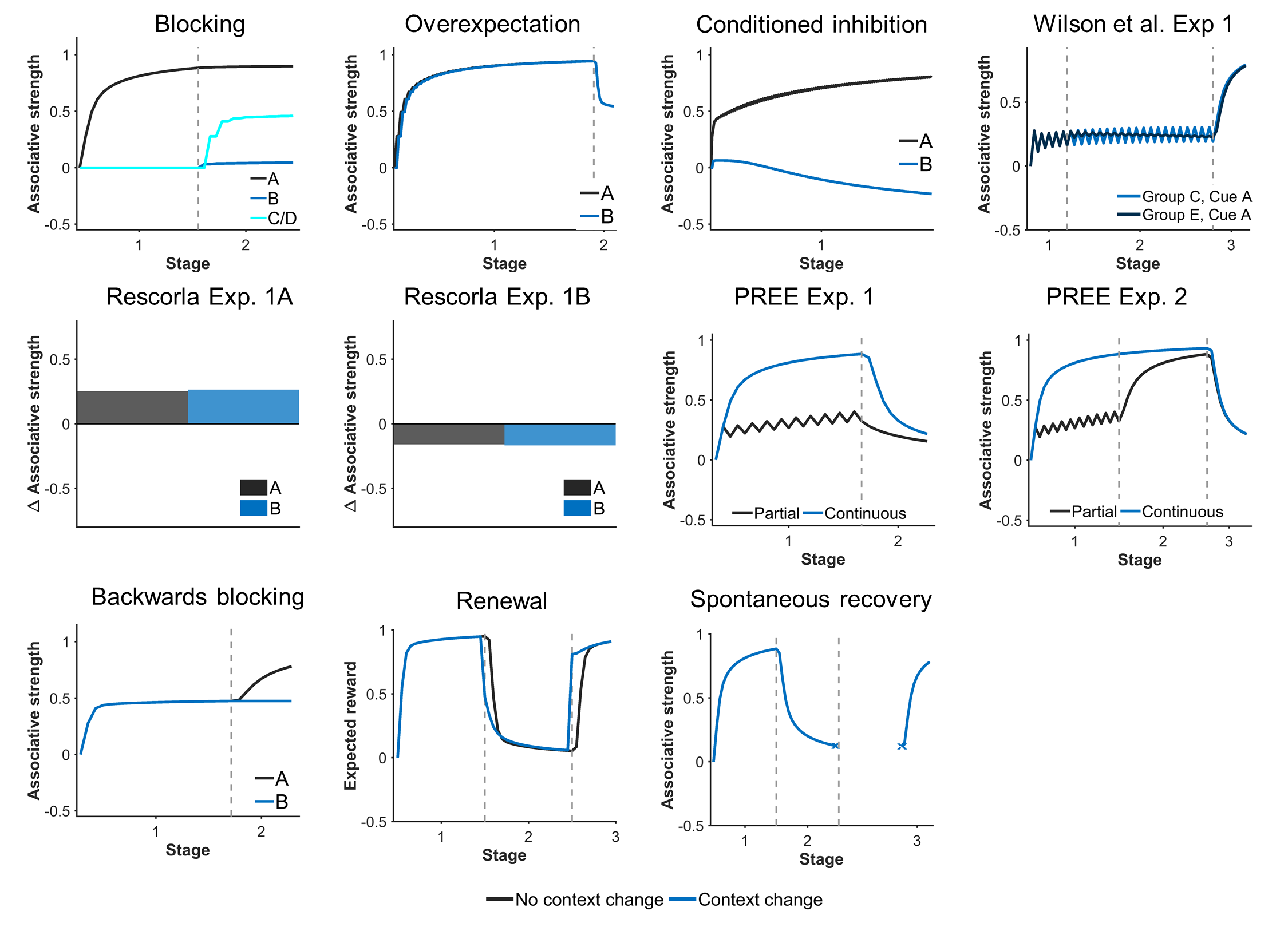

Supplement: S1 Fig — Simulated behavior of the Hybrid model for the same learning experiments in the main text. Gray dashes demarcate experimental stages. (TIF) [file pcbi.1007331.s002.tif]

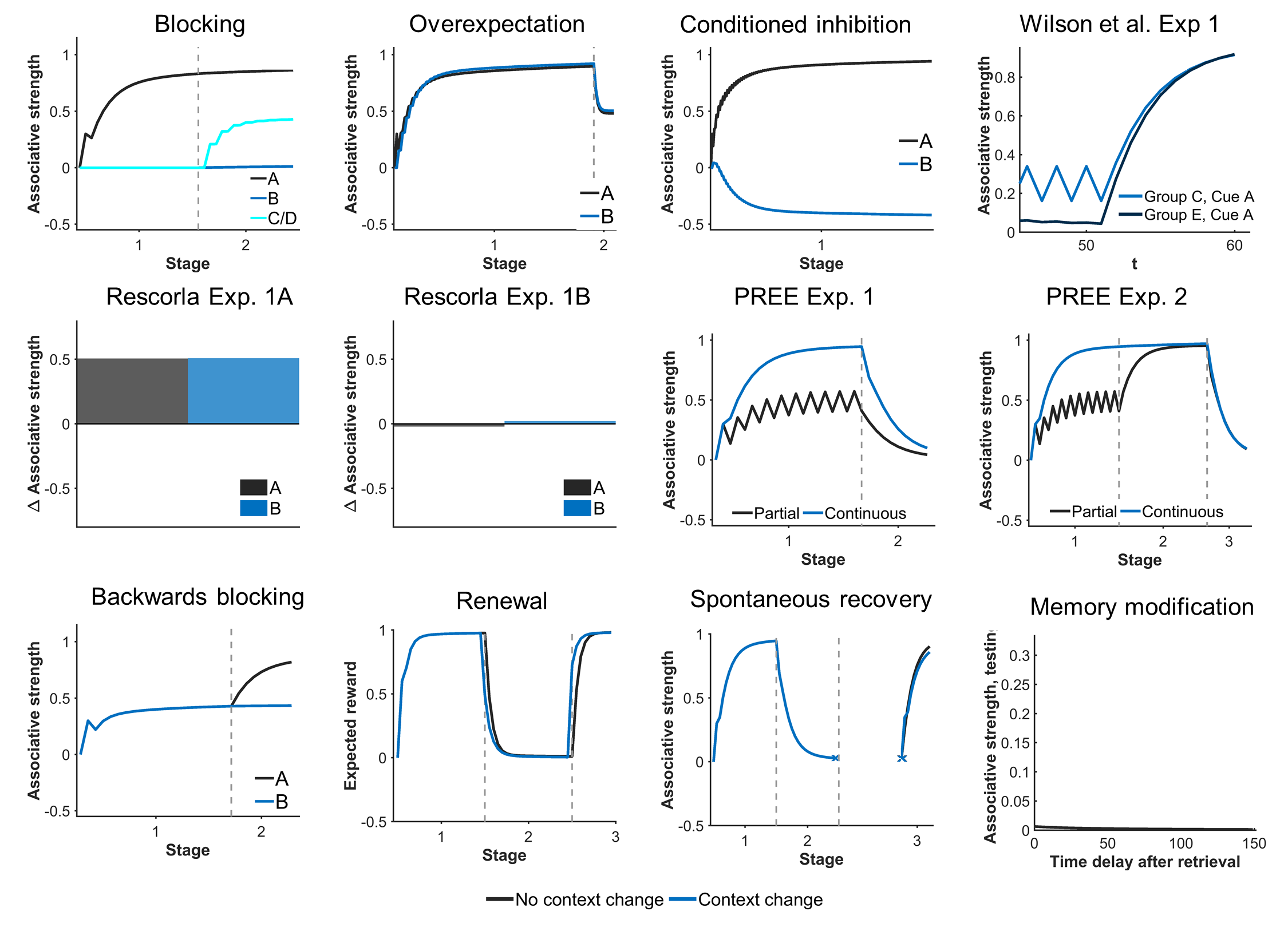

Supplement: S2 Fig — Simulated behavior of Gershman (2017) model with concentration parameter α = 1 for the same learning experiments in the main text. Gray dashes demarcate experimental stages. (TIF) [file pcbi.1007331.s003.tif]
