# Supplementary material for: A flexible and generalizable model of online latent-state learning
Source: PLoS Comput Biol. 2019 Sep 16;15(9):e1007331. doi: 10.1371/journal.pcbi.1007331 (PMC6762208; doi:10.1371/journal.pcbi.1007331)
Supplement: S1 Table — Quantities measured in each simulation experiment in order to test sensitivity of model predictions to changes in parameters. The value of each test quantity determines whether or not a target learning effect is reproduced by the model. (PDF) [file pcbi.1007331.s004.pdf]

#	Experiment	Test quantity
(1)	Blocking	Difference in associative strength of cue C vs cue B at task end
(2)	Overexpectation	Difference in associative strength of cue A at stage 1 end vs stage 2 end
(3)	Conditioned inhibition	Negative of associative strength of cue B at task end
(4)	Backwards blocking	Difference in associative strength of cue B at stage 1 end vs stage 2 end
(5)	Rescorla (2000) 1A	Difference in change in associative strength of cue B vs cue A across stage 2
(6)	Rescorla (2000) 1B	Difference in change in associative strength of cue B vs cue A across stage 2
(7)	Wilson et al (1992) 1	Difference in associative strength of cue A at task end for Group E vs Group C
(8)	PREE Exp 1	Difference in associative strength of cue A in partial reinforcement group vs continuous reinforcement group at start of stage 2 (i.e. extinction)
(9)	PREE Exp 2	Difference in associative strength of cue A in partial reinforcement group vs continuous reinforcement group at start of stage 3 (i.e. extinction)
(10)	Renewal (rapid return)	Difference in expected rewards on second trial of stage 3 (renewal) vs second trial of stage 1 (acquisition)
(11)	Renewal (w/ context)	Difference in expected rewards on second trial of stage 3 (renewal) with visual/spatial context shift vs no context shift
(12)	Spontaneous recovery	Difference in associative strength of a cue on second trial of stage 3 (renewal) with temporal context shift vs no temporal context shift
(13)	Memory modification	Difference in associative strength of a cue on test trial with time delay of 5 after retrieval vs time delay of 1

Table S1. Quantities measured in each simulation experiment in order to test sensitivity of model predictions to changes in parameters. The value of each test quantity determines whether or not a target learning effect is reproduced by the model.
