# Supplementary material for: A flexible and generalizable model of online latent-state learning
Source: PLoS Comput Biol. 2019 Sep 16;15(9):e1007331. doi: 10.1371/journal.pcbi.1007331 (PMC6762208; doi:10.1371/journal.pcbi.1007331)
Supplement: S2 Table — Target quantity as a function of target effect (#1–13) and which parameter was varied, keeping all other parameters fixed. By checking whether each target quantity was positive, we could determine whether the model reproduced one of the target effects listed in the main text. In this table, all parameter sets reproduced target effects. (PDF) [file pcbi.1007331.s005.pdf]

	α_0		β_0		γ		σ_0	
#	0.045	0.055	0.045	0.055	0.045	0.055	0.45	0.55
(1)	0.070	0.083	0.082	0.082	0.083	0.083	0.083	0.091
(2)	0.324	0.327	0.327	0.327	0.327	0.327	0.327	0.330
(3)	0.250	0.250	0.250	-0.250	0.250	0.250	0.250	0.250
(4)	0.067	0.065	0.071	0.071	0.071	0.071	0.071	0.075
(5)	1.000	1.000	1.000	1.000	1.000	1.000	1.000	1.000
(6)	1.000	1.000	1.000	1.000	1.000	1.000	1.000	1.000
(7)	0.090	0.083	0.073	0.092	0.073	0.073	0.073	0.059
(8)	0.308	0.292	0.293	0.293	0.292	0.292	0.292	0.277
(9)	0.003	0.002	0.002	0.002	0.002	0.002	0.002	0.002
(10)	0.150	0.228	0.221	0.262	0.230	0.230	0.230	0.277
(11)	0.095	0.037	0.044	0.014	0.037	0.037	0.037	0.010
(12)	0.044	0.018	0.021	0.007	0.018	0.018	0.018	0.005
(13)	0.002	0.002	0.002	0.002	0.002	0.002	0.001	0.003

Table S2. Target quantity as a function of target effect (#1–13) and which parameter was varied, keeping all other parameters fixed. By checking whether each target quantity was positive, we could determine whether the model reproduced one of the target effects listed in the main text. In this table, all parameter sets reproduced target effects.
